# Comprehensive upstream and downstream regulatory analyses identify miR-675-3p as a potential prognostic biomarker in melanoma

**DOI:** 10.1007/s13577-020-00473-0

**Published:** 2021-01-05

**Authors:** Cai-Chou Zhao, Hao Guo, Ying Wang, Jiu-Hong Li

**Affiliations:** 1 Department of Dermatology, No. 1 Hospital of China Medical University, 155 North Nanjing Street, Heping Distinct, Shenyang, 110001 Liaoning China; 2 Department of Dermatology, Shengjing Hospital of China Medical University, Heping District, Shenyang, 110004 Liaoning China

**Keywords:** Melanoma, miR-675-3p, Prognostic biomarker, Signaling pathways, Bioinformatics

## Abstract

This study assessed miR-675-3p-related regulatory mechanisms in melanoma and the clinical relevance of such regulatory activities. We downloaded miRNA mature strand expression RNA-Seq, phenotypic, and DNA methylation data pertaining to the TCGA Melanoma cohort. Differentially expressed miRNAs (DEMs) between metastatic and primary melanoma patient tissues were then identified, and miR-675-3p expression in melanoma patient peripheral blood was confirmed using the GSE20994 GEO dataset, while its expression in melanoma cell lines was evaluated via qRT-RCR. The clinical and prognostic implications of miR-675-3p in melanoma were assessed, and miR-675-3p target genes were identified using bioinformatics tools. Functional roles of this miRNA were explored via Gene Ontology (GO) and Kyoto Encyclopedia of Genes and Genomes (KEGG) analyses. We identified 3 and 22 miRNAs that were up- and downregulated, respectively, in metastatic melanoma samples relative to primary melanoma samples. Upregulation of miR-675-3p was associated with poorer overall patient survival, tumor histologic grade, and Clark's level. Consistently, miR-675-3p was also overexpressed in the peripheral blood of melanoma patients relative to healthy controls, and in melanoma cell lines relative to control cells. Gene regulatory networks indicated that 32 transcription factors control miR-675-3p expression, and that it, in turn, regulates 10 target genes. KEGG analyses indicated that these genes were associated with cell cycle, transcriptional misregulation in cancer, TGF-beta signaling, and HIF-1 signaling pathways. Gain-of-function assays revealed that miR-675-3p could promote cell proliferation via accelerating cell cycle progression. Western blotting results indicated that miR-675-3p could active TGF-beta and HIF-1 signaling. Through upstream and downstream analyses of miR-675-3p-related regulatory activity, we confirmed that this miRNA participates in key melanoma-related processes and offers value as a prognostic biomarker in melanoma patients.

**Supplementary Information:**

The online version contains supplementary material available at 10.1007/s13577-020-00473-0.

## Introduction

Malignant melanoma is a highly invasive and metastatic disease that is associated with the highest mortality of all forms of skin cancer [1, 2]. When diagnosed at an early stage, melanoma can be readily treated such that over 95% of patients are alive after 5 years. However, in patients with metastatic melanoma, the long-term survival rate is just 5% [3]. The global incidence of melanoma is steadily rising, and it is currently the fifth and seventh most common form of malignancy among males and females, respectively [4]. Treatment options for those with metastatic disease remain limited, and the mechanisms governing the occurrence of such disease remain unclear. While there has been significant progress in the accurate diagnosis of melanoma in recent years, its incidence continues to rise. It is thus urgent that the molecular mechanisms regulating this cancer type be clarified to highlight novel diagnostic or prognostic biomarkers that can be used to guide patient treatment.

MicroRNAs (miRNAs) are short RNAs between 18 and 25 nucleotides long that lack protein-coding functionality [5]. Nonetheless, these miRNAs can control myriad biological processes by specifically binding to the 3′-untranslated region (UTR) of target mRNAs and controlling their expression at the post-transcriptional level [6–8]. Recent improvements in next-generation sequencing technologies have led researchers to identify increasingly large numbers of miRNAs that are dysregulated in the context of cancer, with specific miRNAs functioning as oncogenes or tumor suppressors in a tissue- and disease-specific manner to control cancer progression [9, 10]. To date, however, relatively few studies have focused on clarifying the functional roles of specific miRNAs in the context of melanoma.

Many large gene expression datasets are currently available to the public and can be leveraged to guide new research projects. Gene Expression Omnibus (GEO) datasets store array- and sequence-based genomic data, enabling users to download specifically curated gene expression profiles pertaining to experimental topics of interest [11]. In addition, the Cancer Genome Atlas (TCGA) compiles whole-genome sequencing data and additional information pertaining to mutations, DNA methylation, and copy number variations associated with 33 kinds of human cancer [12]. The goal of this study was, therefore, to leverage these available datasets to identify differentially expressed miRNAs (DEMs) associated with metastatic melanoma, and to understand the upstream and downstream regulatory mechanisms associated with these DEMs as well as their clinical relevance.

## Materials and methods

### Database analyses

We downloaded miRNA mature strand expression RNA-Seq, phenotypic, and DNA methylation 450 k data pertaining to the TCGA Melanoma cohort from these UCSC Xena datasets. RNA-Seq data were then used to identify DEMs between metastatic and primary melanoma samples. The GSE20994 dataset, which contains miRNA expression profiles pertaining to the peripheral blood of 35 melanoma patients and 22 normal controls, was used to assess miR-675-3p expression in blood samples. Phenotypic data and survival outcomes were utilized for Kaplan–Meier survival analyses and chi-squared tests. DNA methylation 450 k data were leveraged to associate the relationship between miR-675-3p expression and the *β* value of the CG locus in the miR-675-3p promoter region.

### DEM identification

Owing to the fact that miRNAs are often expressed at very low levels, we found that several of the samples in the RNA-Seq dataset exhibited empty values pertaining to many miRNAs. As such, excluded all miRNAs that were associated with empty expression values in more than half of the available RNA-Seq analyses. After this exclusion, we screened for DEMs using the R *limma* package with the following cut-off criteria: |fold change (FC)|> 2 and adjusted *p* < 0.05.

### Cell culture

The human A375 malignant melanoma cell line and the human A2058 and 451Lu metastatic melanoma cell lines, as well as the human normal melanocyte PIG1 cell line and human embryonic kidney 293 T (HEK293T) cells, were purchased from the Type culture collection of the Chinese Academy of Sciences (Shanghai, China). A375, A2058, and 451Lu cells were cultured in RPMI-1640 (Hyclone, UT, USA), 293 T cells were cultured in high glucose DMEM (Hyclone) supplemented with 10% FBS, while PIG1 cells were cultured in Medium 254 with Human Melanocyte Growth Supplement (Gibco; Thermo Fisher Scientific) and 5% FBS (Gibco). All cells were grown in a 5% CO_2_ 37 °C incubator, and all media was supplemented with penicillin/streptomycin (Sigma-Aldrich). Prior to collection in the logarithmic phase of growth, cells were rinsed thrice with PBS and harvested using trypsin–EDTA (Solarbio, Beijing, China), after which they were plated into 60 mm tissue culture plates.

### qRT-PCR

TRIzol (Invitrogen, USA) was used to extract total cell RNA based on provided directions, after which a NanoDrop1000 spectrophotometer (Thermo Fisher Scientific, IL, USA) was used to measure RNA concentrations. Reverse transcription was conducted using the Hairpin-it miRNAs qPCR Quantitation Kit (GenePharma, China), with the ABI 7900HT Fast Real-Time PCR system (Applied Biosystems). Threshold cycle (Ct) values for mRNA and miRNA species were normalized to housekeeping genes: GADPH for mRNAs and U6 for mature miRNAs (primers are listed in Table 1).

**Table 1:** Primers and sequence list

Prime name	Sequence (5ʹ–3ʹ)
hsa-miR-675-3p-FO	AACTTGCTGCTGTATGCCCTC
hsa-miR-675-3p-RE	TATGGTTGTTCACGACTCCTTCAC
U6 snRNA-FO	CGCTTCGGCAGCACATATAC
U6 snRNA-RE	TTCACGAATTTGCGTGTCATC
hsa-miR-675-3p mimic-sense	CUGUAUGCCCUCACCGCUCA
hsa-miR-675-3p mimic-antisense	AGCGGUGAGGGCAUACAGUU
mimic NC-sense	UUCUCCGAACGUGUCACGUTT
mimic NC-antisense	ACGUGACACGUUCGGAGAATT
IGF1R-FO	TGCTGACCTCTGTTACCTCTCCAC
IGF1R-RE	GTCTTCTCACACATCGGCTTCTCC
EGR1-FO	AGCAGCAGCAGCACCTTCAAC
EGR1-RE	CCACCAGCACCTTCTCGTTGTTC
OPCML-FO	ATCTCTGACATCAAGCGAGACC
OPCML-RE	CTTCTGACCGACTGAAACACC
GAPDH-FO	CAGCCTCAAGATCATCAGCAAT
GAPDH-RE	ATGAGTCCTTCCACGATACCAA

### Transcription factor (TF) and target gene prediction

The TransmiR v2.0 [13] and hTFtarget [14] tools were employed to predict TFs likely to regulate miR-675-3p expression. The beta-model score[15] used to identify putative TF–target relationships was as follows:

$$S_{{\text{g}}} = \sum\limits_{i = 1}^{k} {{\text{e}}^{ - (0.5 + 4\Delta i)} } ,$$

where *S*_g_ corresponds to the beta-model score, which is the sum of the weighted scores of peaks proximal to the transcriptional start site (TSS) of a given gene g; *k* is the number of binding sites within 50 kb of this TSS; Δ*i* is the distance between the summit of peak *i* and the TSS (normalized to 50 kb, such that values of 0.04 and 1 correspond to 2 and 50 kb, respectively).

Putative miR-675-3p target genes were identified using the TargetScan Human v7.1 [16], miRDB [17], and miRTarBase [18] databases, with targets that were predicted by all three of these tools being utilized in downstream analyses.

### Gene functional enrichment analyses

To assess biological roles and cancer-related pathways associated with the miR-675-3p regulatory network, we conducted GO, KEGG, and network topology-based analyses with the WEB-based GEne SeT AnaLysis Toolkit [19].

### miR-675-3p mimic transfection

The human miR-675-3p mimic and Negative control constructs were designed and provided by GenePharma (GenePharma, China). Cells that were 30–50% confluent were transfected with miRNAs using Lipofectamine 2000 (Invitrogen, USA) according to the manufacturer’s protocol. The mimic sequences and primers used in this study are shown in Table 1.

### Western blotting

The total proteins were extracted using a RIPA assay kit. Total protein was quantified by BCA Protein Assay Kit (Beyotime, China). Subsequently, the proteins were separated via SDS-PAGE and then transferred onto a polyvinylidene fluoride (PVDF) membrane (Pall, USA). The membranes were blocked with the QuickBlock™ Blocking Buffer (P0252; Beyotime Biotechnology) at room temperature for 15 min at room temperature, and blots were then incubated at 4 °C with the following diluted primary rabbit anti-human antibodies: anti-IGF1R (1:3000, K106546P; Solarbio), anti-EGR1 (1:600, D120585; BBI), anti-OBCAL (1:1000, DF8584; Affinity), anti-TGF beta1 (1:1000, AF1027; Affinity), anti-TGF beta2 Ab (1:1000, AF0260; Affinity), anti-Smad2/3 (1:1000, AF6367; Affinity), anti-Smad4 (1:1000, AF5247; Affinity), anti-HIF1A (1:1000, AF1009; Affinity), and anti-beta Actin (1:5000, AF7018; Affinity), followed by horseradish peroxidase (HRP)-labeled goat anti-rabbit immunoglobulin G (IgG) H&L (1:5000; ZB-2301; ZSG-BIO) for 2 h at room temperature. After washing three times in TBST, bands were visualized utilizing BeyoECL Plus Moon (P0018S; Beyotime Biotechnology). The immunoblotting results were analyzed using the Image J software.

### Cell counting Kit-8 (CCK8)

Cells transfected with miR-675-3p mimic or NC constructs were digested with trypsin and seeded (3 × ${10}^{3}$ /well) in 96-well plates. Cell proliferative activity at different time points (24 h, 48 h, 72 h) was detected with a CCK8 assay kit (TargetMOL, USA). Cells were treated with 10 µL/well of CCK8 reagent and incubated for 2 h, and absorbance was measured at 450 nm via a microplate reader (BioTek, USA).

### Flow cytometry

Propidium iodide (PI) single staining: cells (1 × ${10}^{6}$) were trypsinized and resuspended to obtain single-cell suspensions at 24 h post-transfection. Detached cells were fixed overnight at 4 °C in 70% ethanol, and were then stained with propidium iodide (Cell Cycle Detection kit; KeyGen) and analysed with a FACScan flow cytometer (BD Biosciences, USA) and the ModFit LT v3.3 software (Verity Software House, USA).

### Dual-luciferase reporter assays

Based on the miRNA databases (microRNA.org, miRDB, and TargetScan databases), qRT-PCR and Western blotting analysis, OBCML was identified as the most likely target of miR-675-3p. As such, we cloned the WT or mutant OPCML 3′-UTR into the pmirGLO luciferase reporter vector (Promega, USA). For luciferase assays, these reporter plasmids were co-transfected with miR-675-3p mimic or control constructs into HEK293T cells. At 24 h post-transfection, cells were lysed and luciferase expression was measured using the Dual-luciferase assay system (Promega, USA) based upon the manufacturer’s protocol. Renilla luciferase (Rluc) was normalized based upon firefly luciferase (Luc) activity. Three independent experiments were performed in duplicate.

### Statistical analysis

Comparisons between groups were made using unpaired or paired Student’s *t* tests, as appropriate. Pearson’s chi-squared tests were used to evaluate relationships between miR-675-3p expression levels and melanoma patient clinicopathological features, while Pearson correlation analyses were employed to gauge the association between miR-675-3p expression and the DNA methylation level *β* value. Kaplan–Meier analyses and log-rank tests were used to assess patient overall survival (OS), with patients being separated into miR-675-3p-low or -high expression groups according to optimal expression cut-off values calculated based upon survival outcomes using X-tile [20]. SPSS 22.0 (IL, USA) was used for all statistical testing, with *p* < 0.05 as the significance threshold.

## Results

### miR-675-3p is upregulated in melanoma cell lines, tissues, and blood

**Fig. 1:**
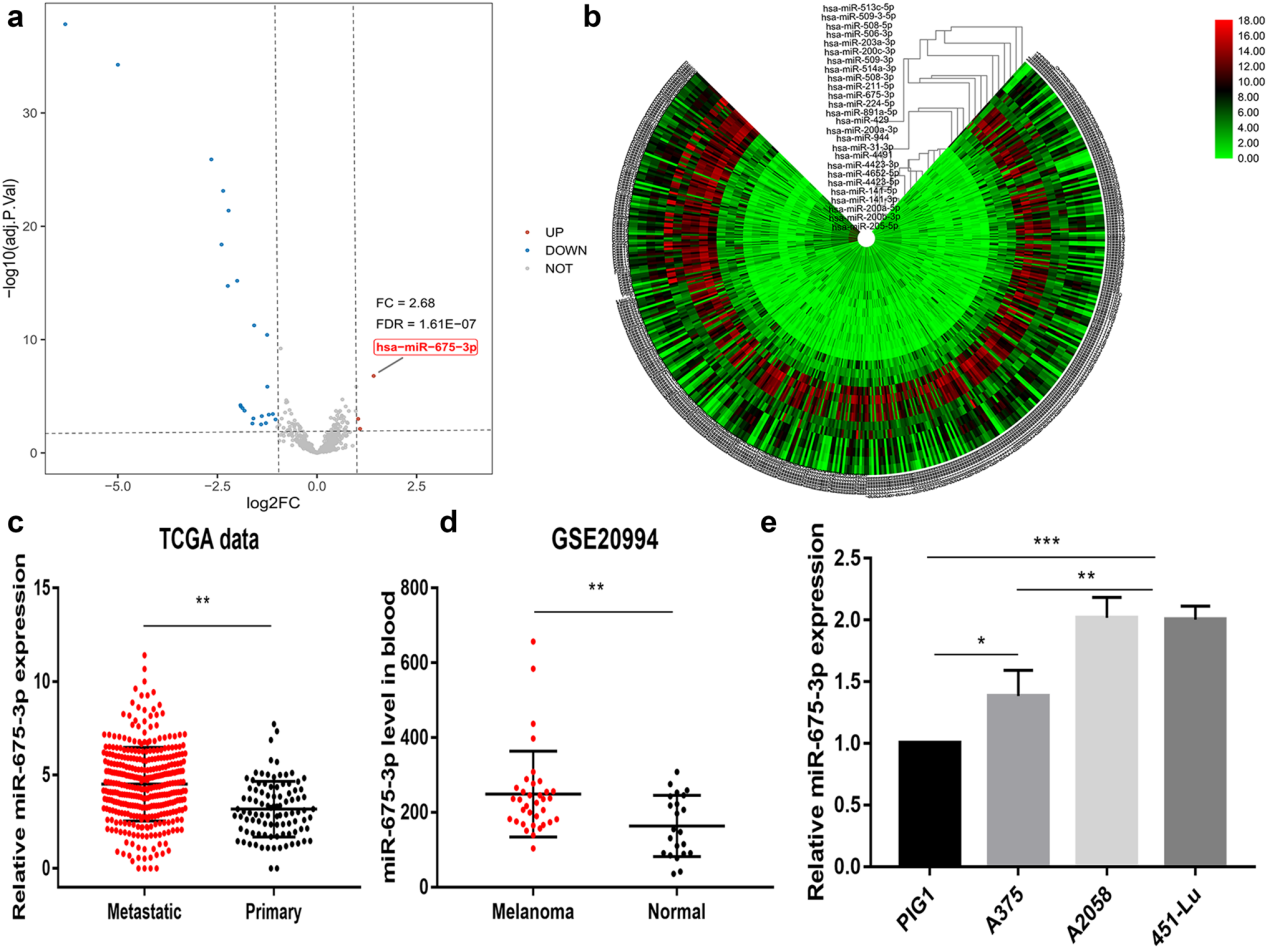
miR-675-3p is upregulated in melanoma. **a** In total, 3 upregulated and 23 downregulated miRNAs were identified in metastatic melanomas samples. **b** A hierarchical clustering heat map representing DEMs in 353 metastatic and 97 primary melanoma tissues was constructed. **c** miR-675-3p was upregulated in metastatic melanoma samples relative to primary tissues in the TCGA miRNA-Seq database. **d** Relative to healthy control samples, miR-675-3p was upregulated in the peripheral blood of melanoma patients in the GSE20994 dataset. **e** miR-675-3p is upregulated in metastatic and primary melanoma cell lines. *p < 0.05, **p < 0.01 and ***p < 0.001

We began by identifying DEMs associated with metastatic melanoma by evaluating a miRNA-Seq expression dataset containing 353 metastatic melanoma and 97 primary melanoma tissue samples. In total, we identified 3 and 23 DEMs that were significantly up- and down-regulated in metastatic melanoma samples, respectively (|FC|> 2; adjusted *p* < 0.05) (Fig. 1a and Table 2). Hierarchical clustering heatmaps were additionally used to visualize DEMs associated with metastatic and primary disease (Fig. 1b). Of the identified Dems, miR-675-3p was the most significantly upregulated in metastatic tissues (FC = 2.68) relative to primary tissues (Fig. 1c). We then explored the expression of this miRNA in melanoma patient peripheral blood and cell lines. Relative to normal control samples, we found that miR-675-3p was significantly upregulated in the peripheral blood of melanoma patients in the GSE20994 dataset relative to healthy controls (Fig. 1d). Additional qRT-PCR assays similarly confirmed that miR-675-3p is upregulated in both metastatic and primary melanoma cell lines (Fig. 1e). Together, these data suggest that miR-675-3p may play an oncogenic role in melanoma.

**Table 2:** The DEMs identified from TCGA miRNA-Seq data

Up-regulated miRNAs	logFC		Adjusted *p* value	Down-regulated miRNAs	logFC		Adjusted *p* value
hsa-miR-675-3p	1.42	1.61E−07		hsa-miR-205-5p	− 6.32	1.48E−38	
hsa-miR-4491	1.02	9.5E−04		hsa-miR-203a-3p	− 5.0	5.58E−35	
hsa-miR-4652-5p	1.06	7.1E−03		hsa-miR-200c-3p	− 2.65	1.26E−26	
				hsa-miR-141-5p	− 2.39	4.09E−19	
				hsa-miR-200b-3p	− 2.35	7.54E−24	
				hsa-miR-141-3p	− 2.24	1.84E−15	
				hsa-miR-200a-5p	− 2.22	4.14E−22	
				hsa-miR-944	− 2.0	6.51E−16	
				hsa-miR-508-5p	− 1.92	6.07E−05	
				hsa-miR-514a-3p	− 1.91	7.92E−05	
				hsa-miR-508-3p	− 1.87	0.000112	
				hsa-miR-509-3p	− 1.82	0.000188	
				hsa-miR-211-5p	− 1.62	0.002545	
				hsa-miR-506-3p	− 1.60	0.000907	
				hsa-miR-200a-3p	− 1.58	5.58E−12	
				hsa-miR-509-3-5p	− 1.40	0.003044	
				hsa-miR-891a-5p	− 1.39	0.000563	
				hsa-miR-513c-5p	− 1.28	0.002328	
				hsa-miR-224-5p	− 1.25	3.83E−11	
				hsa-miR-429	− 1.25	1.43E−06	
				hsa-miR-4423-5p	− 1.21	0.000419	
				hsa-miR-4423-3p	− 1.11	0.000371	
				hsa-miR-31-3p	− 1.04	0.001113	

### Assessment of the clinical implications of miR-675-3p expression in melanoma

**Fig. 2:**
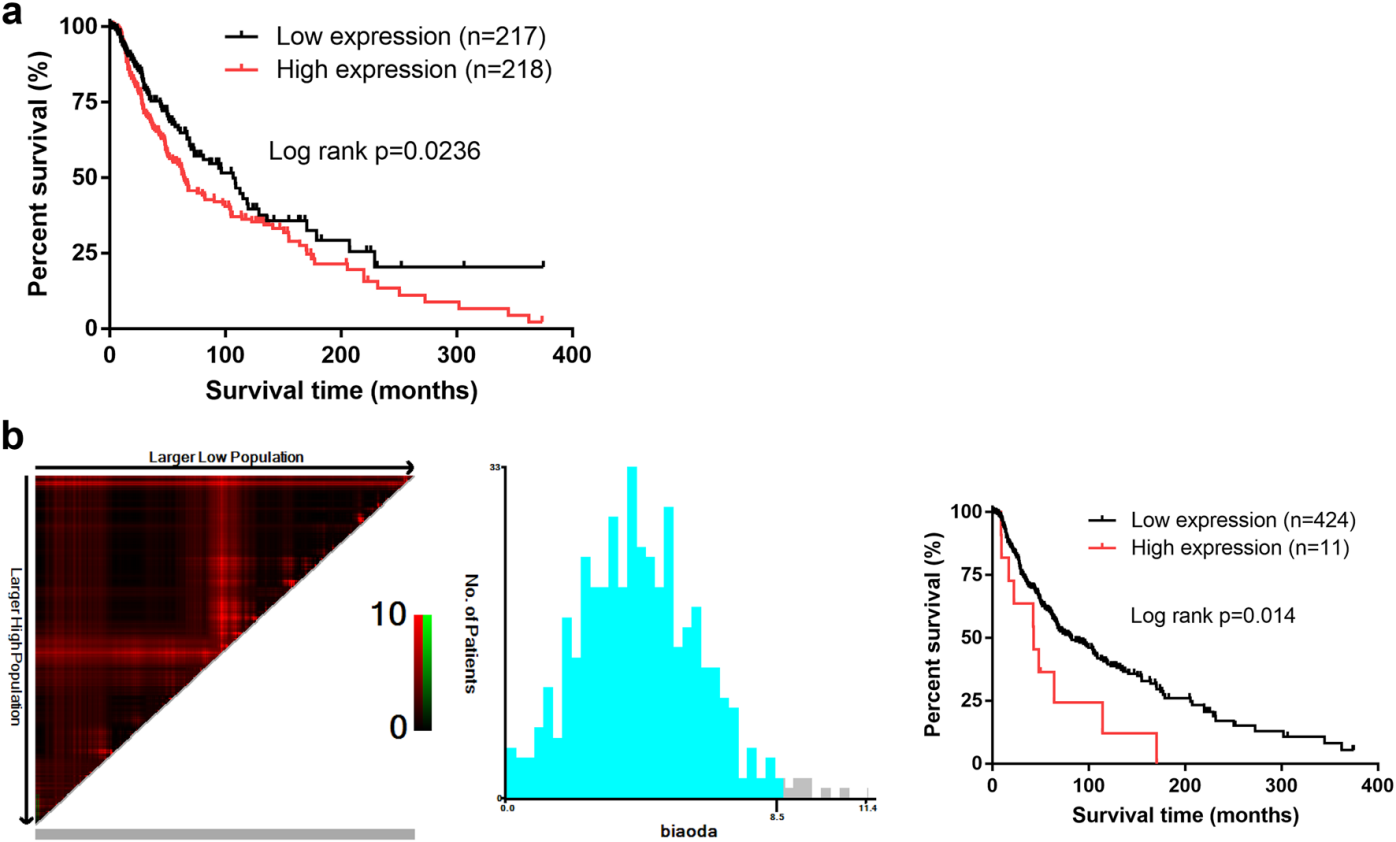
miR-675-3p may represent a prognostic biomarker in melanoma. **a** Kaplan–Meier analyses in which melanoma patients were separated based upon median miR-675-3p expression levels revealed that high miR-675-3p expression was associated with poorer OS. **b** X-tile was used to calculate an optimal miR-675-3p expression cut-off value, which revealed that elevated expression of this miRNA was associated with poorer melanoma patient OS

We next conducted analyses of the prognostic relevance of miR-675-3p by separating patients for whom overall survival (OS) data was available into miR-675-3p-high and -low groups based upon the median expression level of this miRNA. We found that patients with elevated miR-675-3p expression levels exhibited poorer OS (Fig. 2a). We then used X-tile to calculate an optimal miR-675-3p expression cut-off value of 8.5 that was subsequently used to again stratify patients into miR-675-3p-high or -low expression groups. This analysis again confirmed that high expression of this miRNA was associated with decreased OS in melanoma patients (Fig. 2b). When Pearson’s chi-squared analyses were conducted to explore the relationship between miR-675-3p expression and clinicopathological features, we found that elevated expression of this miRNA was correlated with tumor histologic grade and Clark’s level, whereas this expression was unrelated to age, gender, TNM stage, family history of cancer, pathologic_M, pathologic_T, or pathologic_N stage (Table 3). Together, these data suggest that miR-675-3p may offer value as a clinically important prognostic biomarker in patients with melanoma.

**Table 3:** Correlation between miR-675-3p expression and clinicopathological features

Variables	Total (*N* = 230)	miR-675-3p expression		*p* value
		High (*N* = 115)	Low (*N* = 115)	
Age (year)				
< 65	120 (52.2%)	61 (53.0%)	59 (51.3%)	0.792
≥ 65	110 (47.8%)	54 (47.0%)	56 (48.7%)	
Gender				
Male	144 (62.6%)	68 (59.1%)	76 (66.1%)	0.276
Female	86 (37.4%)	47 (40.9%)	39 (33.9%)	
Family history of cancer				
No	133 (57.8%)	66 (57.4%)	67 (58.3%)	0.713
Yes	67 (29.1%)	32 (27.8%)	35 (30.4%)	
Unknown	30 (13.0%)	17 (14.8%)	13 (11.3%)	
TNM stage				
I	57 (24.8%)	31 (27.0%)	26 (22.6%)	0.531
II	78 (33.9%)	35 (30.4%)	43 (37.4%)	
III	89 (38.7%)	47 (40.9%)	42 (36.5%)	
IV	6 (2.6%)	2 (1.7%)	4 (3.5%)	
Histologic grade				
G1–G2	155 (67.4%)	70 (60.9%)	85 (73.9%)	0.035*
G3–G4	75 (32.6%)	45 (39.1%)	30 (26.1%)	
Pathologic_M				
M0	224 (97.4%)	113 (98.3%)	111 (96.5%)	0.408
M1	6 (2.6%)	2 (1.7%)	4 (3.5%)	
Clark's level				
I–III	47 (20.4%)	17 (14.8%)	30 (26.1%)	0.034*
IV–V	183 (79.6%)	98 (85.2%)	85 (73.9%)	
Pathologic_N				
N0–N1	135 (75.2%)	69 (60.0%)	66 (57.4%)	0.688
N2–N4	95 (24.8%)	46 (40.0%)	49 (42.6%)	
Pathologic_T				
T1–T2	82 (35.7%)	45 (39.1%)	37 (32.2%)	0.271
T3–T4	148 (64.3%)	70 (60.9%)	78 (67.8%)	

*indicates statistically significant. The high expression of miR-675-3p was correlated with tumor histologic grade and Clark’s level

### The relationship between DNA methylation, miR-675-3p expression, and clinical features

**Fig. 3:**
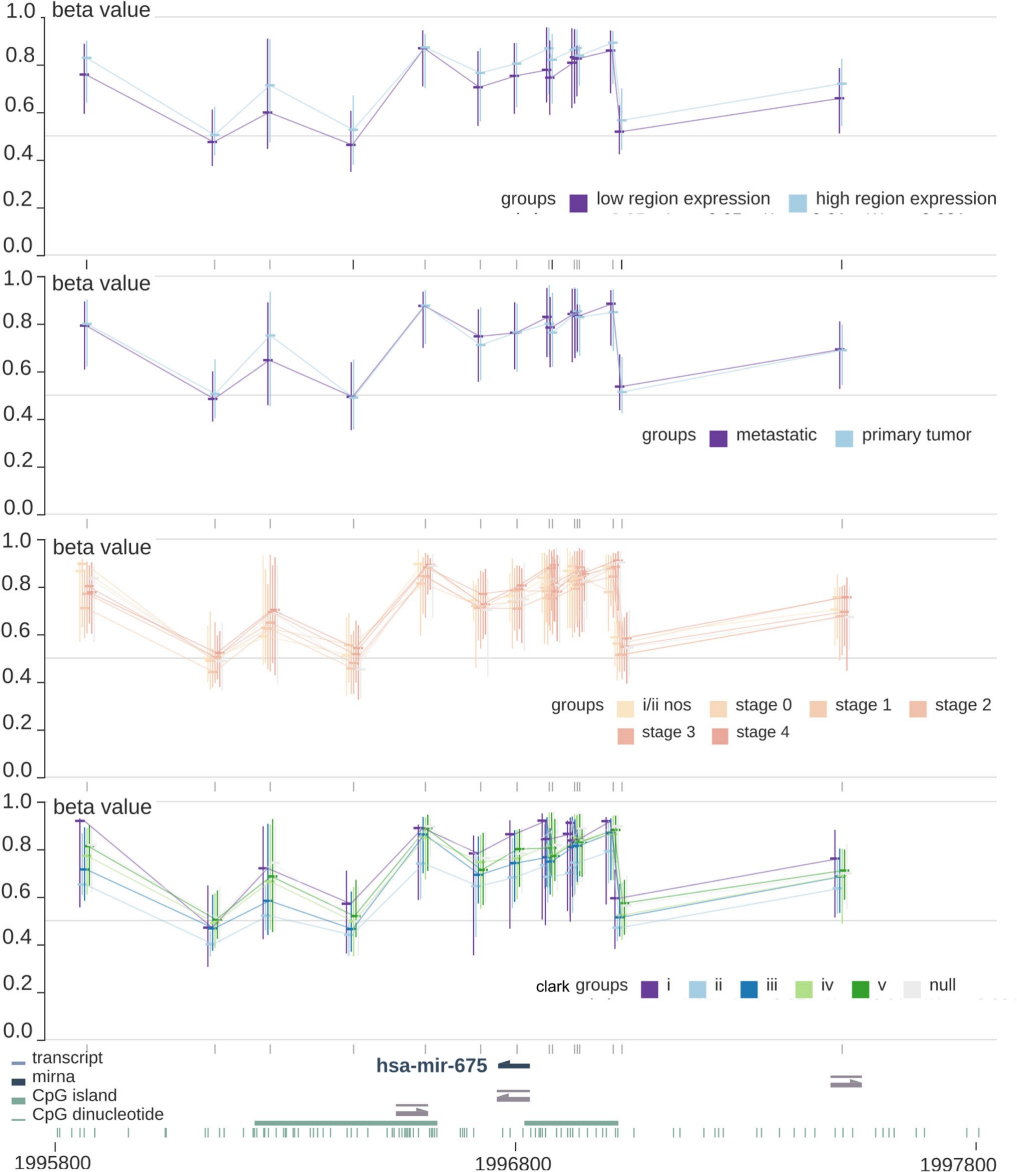
The relationship between DNA methylation, miR-675-3p expression, and clinical features. A beta value was calculated for each CpG target with Illumina's Bead Studio software with the Methylation Module v3.2. All *p* values were greater than 0.05 indicated the level of DNA methylation level was not significantly associated with miR-675-3p expression (low/high), sample type (primary/metastatic), tumor stage (simplified), or Clark’s level

In light of the apparent oncogenic role of miR-675-3p in melanoma, we next assessed the upstream mechanisms governing its regulation. We began using the MEXPRESS database [21] to evaluate the relationship between DNA methylation and miR-675-3p expression. We detected no significant differences between DNA methylation status and miR-675-3p expression level (low/high), sample type (primary/metastatic), tumor stage (simplified), or Clark’s level (Fig. 3). Pearson’s correlation analyses between miR-675-3p expression levels and the *β* values corresponding to 9 relevant CG loci in the promoter region (cg03175030, cg07342901, cg14937069, cg15269875, cg15963714, cg19943238, cg21167159, cg25852472, and cg26857192) revealed negative correlations for all tested loci, but these trends did not achieve statistical significance (Supplementary Fig. 1).

### Functional analyses of the up- and downstream miR-675-3p regulatory network

**Fig. 4:**
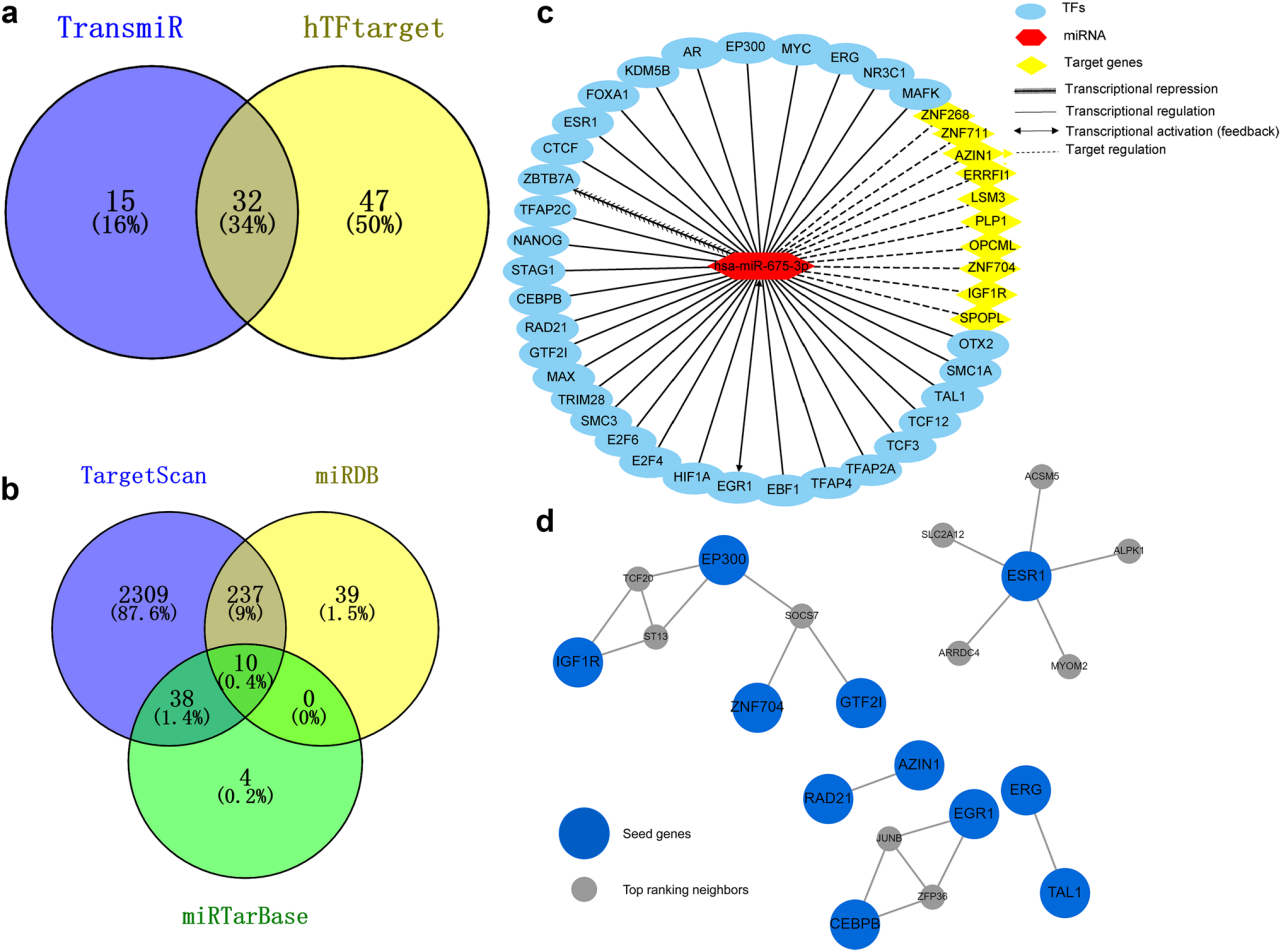
The up- and downstream miR-675-3p regulatory network. **a** In total, 32 overlapping TFs were predicted by both TransmiR and hTFtarget to regulate miR-675-3p expression. **b** In total, TargetScan Human v7.1, miRDB, and miRTarBase identified 10 putative miR-675-3p target genes. **c** A comprehensive up- and downstream miR-675-3p regulatory incorporating these 32 TFs and 10 target genes was constructed. **d** A network topology-based analysis revealed a close sub-network between seed genes and top-ranking neighbors

We next sought to identify TFs involved in the upstream regulation of miR-675-3p expression. The TransmiR and hTFtarget databases identified 47 and 79 putative TFs regulating the expression of this miRNA, respectively, of which 32 were found to overlap between both datasets and were thus considered to be probably transcriptional regulators of miR-675-3p expression (Fig. 4a). In total, 10 overlapping miR-675-3p target genes were predicted using TargetScan Human v7.1, miRDB, and miRTarBase (Fig. 4b). We then constructed a comprehensive miR-675-3p regulatory network incorporating these 32 TFs and 10 target genes (Fig. 4c). Notably, this network suggested that EGR1 may play a feedback role in the regulation of miR-675-3p expression, while ZBTB7A may repress the expression of this miRNA.

**Fig. 5:**
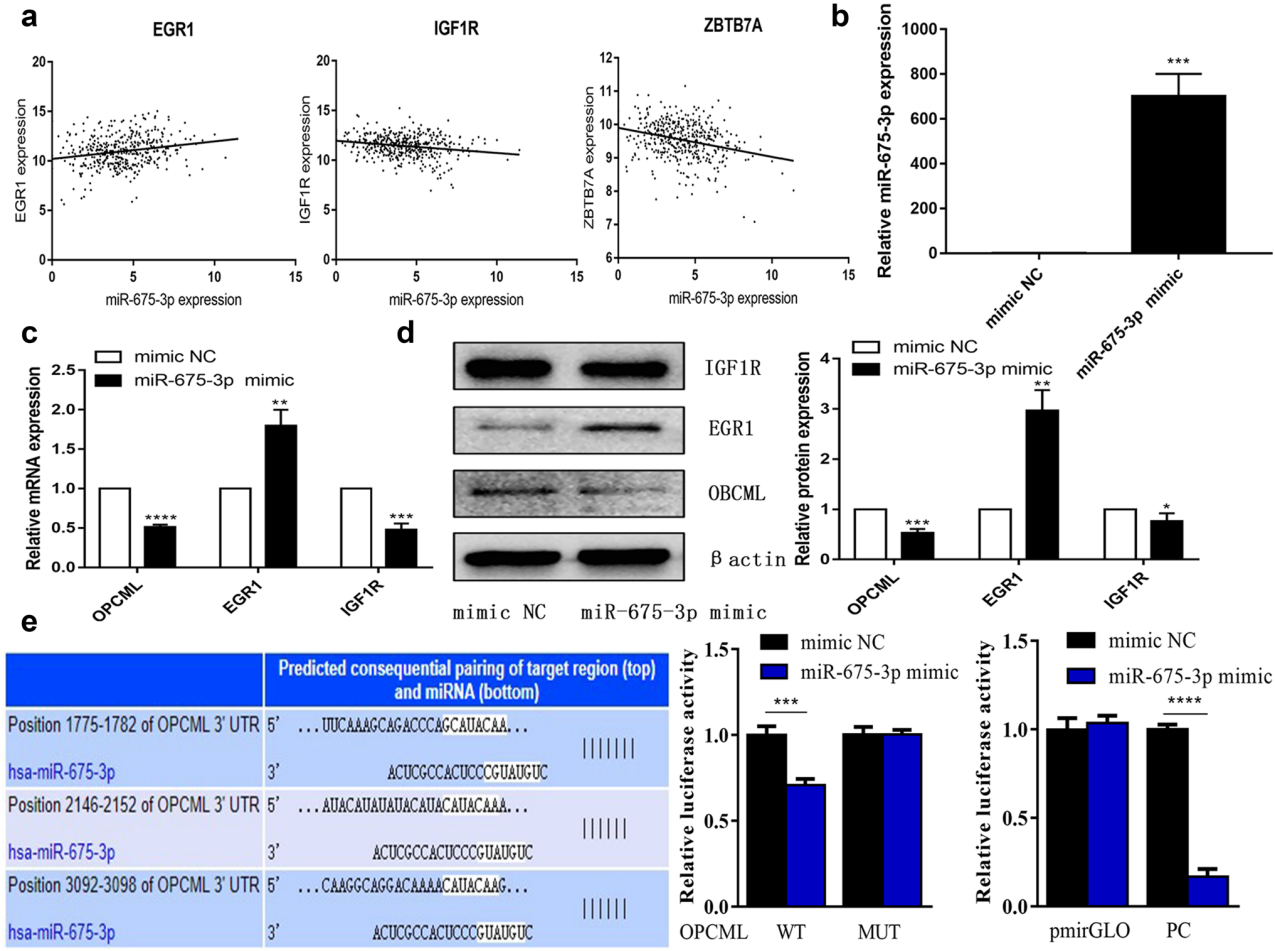
Identification of miR-675-3p target genes. **a** Pearson’s correlation analyses showed that miR-675-3p was significantly positively correlated with ERG1 and negatively correlated with ZBTB7 and IGF1R expression in the TCGA database. **b** Validation of transfection efficiency. **c** Relative target gene mRNA expression. **d** Relative target gene protein expression. **e** OPCML was targeted by miR-675-3p in a dual luciferase reporter experiment

In addition, TFs and target genes were utilized in functional enrichment analyses to further understand the potential biological role of miR-675-3p. A network topology-based analysis revealed a sub-network containing seed genes and top-ranking neighbors (Fig. 4d). Pearson’s correlation analyses showed that miR-675-3p was significantly positively correlated with ERG1 and negatively correlated with ZBTB7 and IGF1R expression in the TCGA database (Fig. 5a). Then, we utilized melanoma cell lines to test the impact of miR-675-3p mimic transfection on the expression of ERG1, IGF1R, and OPCML targets. qRT-PCR was used to verify transfection efficiency (Fig. 5b). Relative to negative control samples, we found that EGR1 was significantly upregulated, while IGF1R and OPCML were significantly down-regulated at the mRNA and protein levels (Fig. 5c, d). Based on the miRNA databases (TargetScan, miRDB and miRTarBase databases), qRT-PCR, and Western blotting, OBCML was identified as the most likely target of miR-675-3p. As such, we confirmed OPCML was targeted by miR-675-3P through these dual-luciferase reporter experiments (Fig. 5e).

**Fig. 6:**
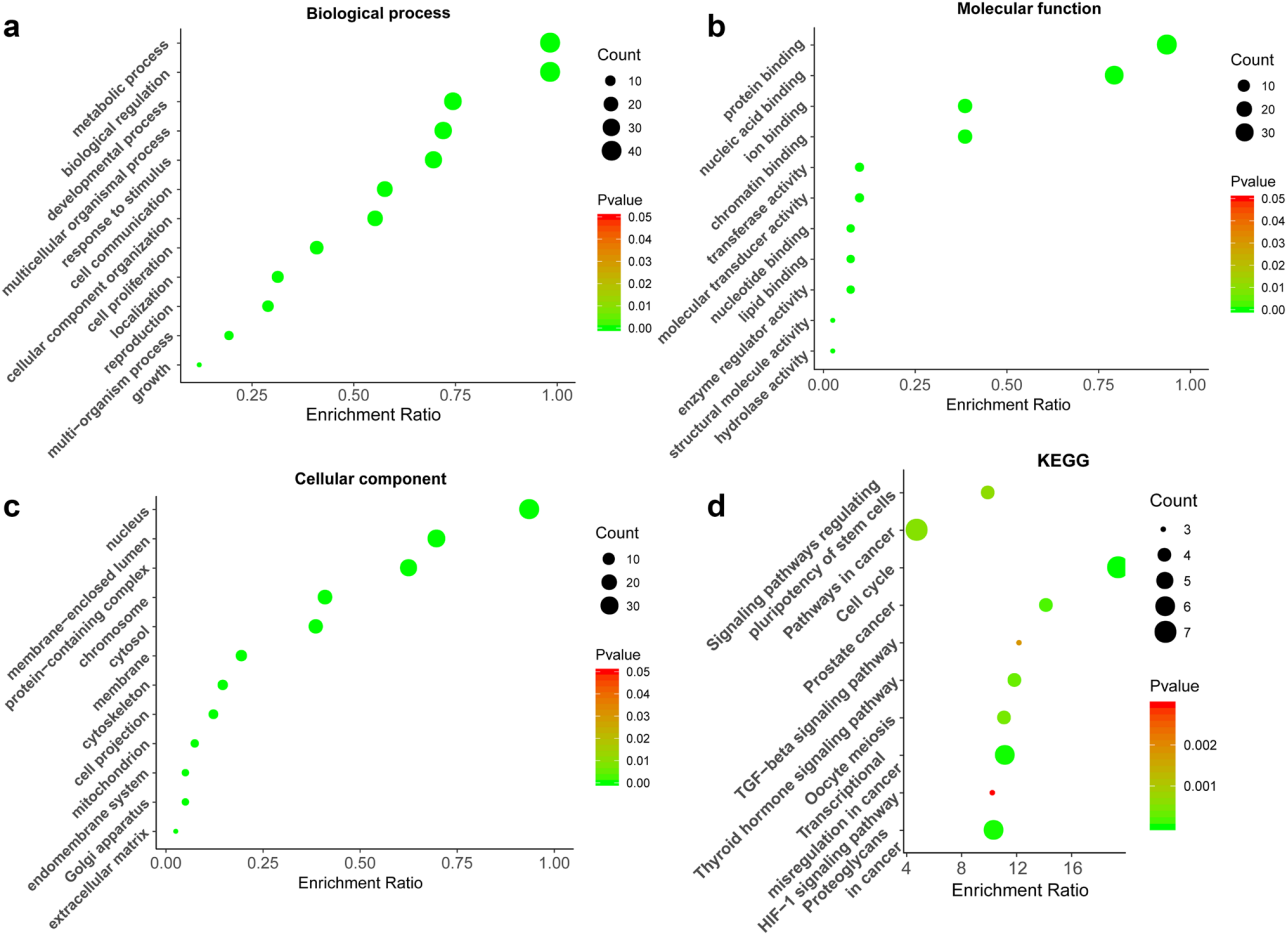
GO and KEGG enrichment analyses. **a**–**c** GO enrichment analyses pertaining to biological processes, molecular functions, and cellular components. **d** A KEGG pathway analysis suggested that miR-675-3p may regulate the cell cycle, transcriptional misregulation in cancer, TGF-beta, and HIF-1 signaling pathways

**Fig. 7:**
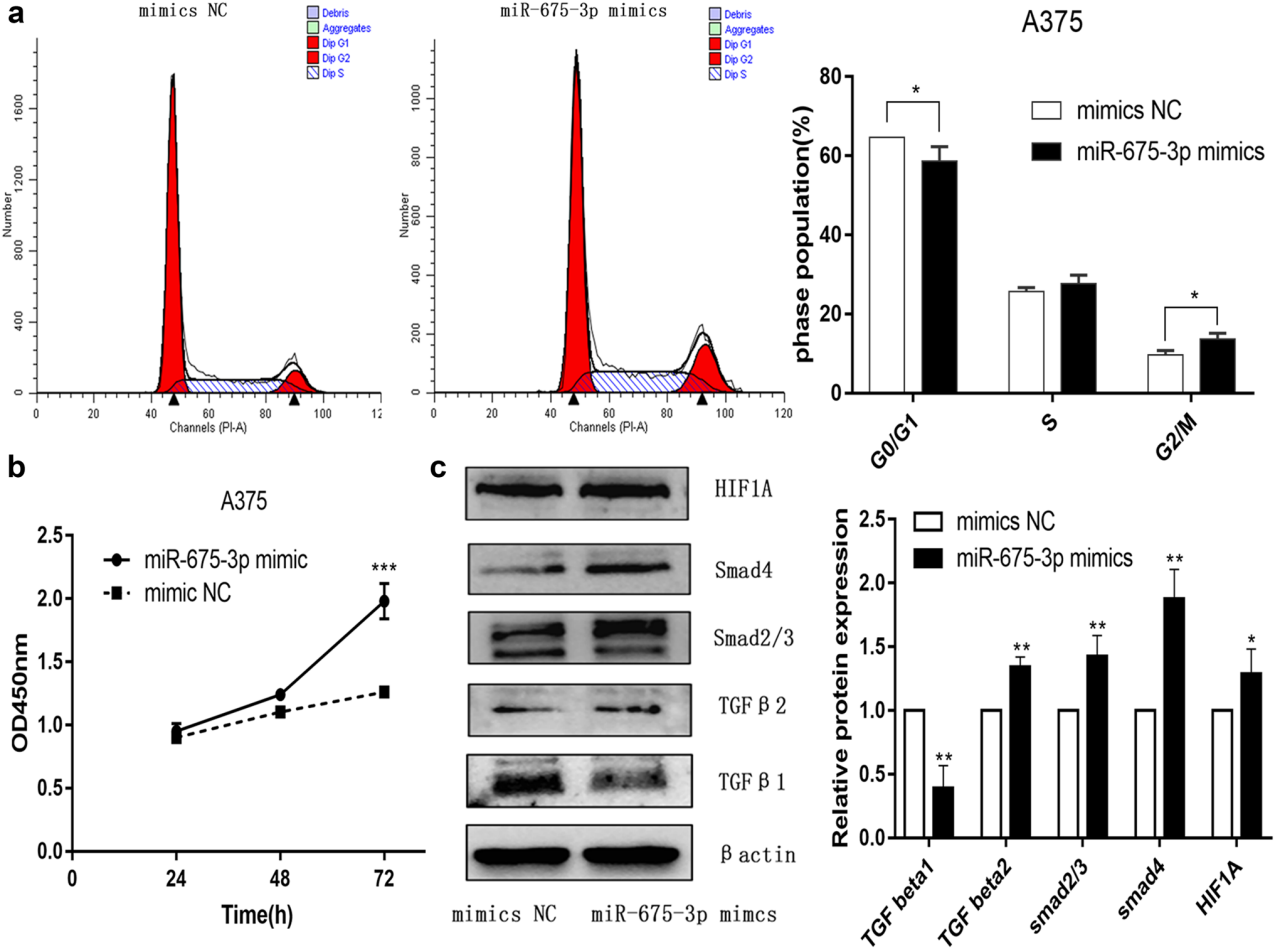
miR-675-3p may promote cell proliferation, regulate the cell cycle, and activate the TGF-β, HIF-1 signaling pathways. **a** miR-675-3p facilitated the G0/G1–G2/M transition in human melanoma A375 cells, **b** miR-675-3p promoted cell proliferation. **c** The TGFβ2, Smad2/3, Smad4, and HIF1A protein levels in the TGF-β/SMAD and HIF-1 signaling pathways were significantly higher, while TGF β1 was decreased relative to the negative control group

GO analyses suggested that these genes participate in key biological processes and molecular functions related to carcinogenesis (Fig. 6a–c), with KEGG analyses further suggesting that miR-675-3p may regulate the cell cycle, transcriptional misregulation in cancer, TGF-beta, and HIF-1 signaling pathways (Fig. 6d). A CCK8 assay demonstrated that miR-675-3P could promote the proliferation of A375 cells (Fig. 7b). Cell cycle analysis by flow cytometry revealed that miR-675-3p mimic decreased the percentage of cells in the G0/G1 phase and increased the frequency of cells in the G2/M phase (Fig. 7a), which suggested that miR-675-3p facilitated the G0/G1–G2/M transition in human melanoma A375 cells. To explore whether miR-675-3p acts as a key modifier affecting the TGF-β and HIF-1 signaling pathways, we upregulated miR-675-3P levels in melanoma cells by transfecting them with a miR-675 mimic construct. Western blotting (Fig. 7c) results revealed that the TGFβ2, Smad2/3, Smad4, and HIF1A protein levels associated with the TGF-β/SMAD and HIF-1 signaling pathways were significantly higher, whereas TGF β1 levels were decreased relative to the negative control group. These results indicated that miR-675-3p positively activates the TGF-β2/SMAD and HIF-1 signaling pathways.

## Discussion

Herein, we identified DEMs that were differentially expressed between metastatic and primary melanoma patient tissues by analyzing the TCGA melanoma miRNA RNA-Seq dataset. Through these analyses, we determined that miR-675-3p was significantly upregulated in metastatic tissues. Kaplan–Meier survival analyses additionally revealed that higher miR-675-3p expression levels were associated with poorer OS in melanoma patients, and chi-squared tests revealed that miR-675-3p overexpression was significantly associated with histologic grade and Clark's level. Consistent with these findings, we determined that miR-675-3p was upregulated in melanoma patient peripheral blood samples and melanoma cell lines. Together, all of these results indicated that miR-675-3p may play an oncogenic role in melanoma.

The miR-675 precursor is encoded on chromosome 11p15.5, and can generate two mature miRNAs (miR-675-5p and miR-675-3p). There is mounting evidence that miR-675-3p serves as an oncogene in a range of cancer types. For example, Xiao et al. determined that miR-675-3p overexpression in esophageal squamous cell cancer cells is linked to enhanced migratory and invasive activity associated with alterations in epithelial–mesenchymal transition marker levels [22]. Furthermore, miR-675-3p was shown to target the DMTF1 3′-UTR in colorectal cancer and to thereby promote enhanced tumor cell proliferation [23]. There is further evidence that miR-675-3p can regulate non-neoplastic diseases, including osteoarthritis [24] and pulmonary arterial hypertension [25]. The specific role of miR-675-3p in the development and progression of melanoma remains unclear. Herein, we determined that miR-675-3p was upregulated in melanoma cell lines, tissues, and blood, and that it may play an oncogenic role in this cancer type.

We additionally explored the up- and downstream regulatory mechanisms associated with the expression of miR-675-3p in melanoma. Altered DNA methylation is frequently associated with the dysregulation of a range of cancer-associated genes and miRNAs [26, 27]. We, therefore, hypothesized that miR-675-3p dysregulation may be induced by aberrant DNA promoter methylation. However, when we analyzed beta values of all CG loci in the miR-675-3p promoter region, we did not detect any significant relationship between miR-675-3p expression levels and methylation status. We further detected no significant associations between DNA methylation level and miR-675-3p expression group (low/high), sample type (primary/metastatic), tumor stage (simplified), or Clark’s level. These results thus suggested that DNA methylation is not associated with miR-675-3p dysregulation.

We next assessed the TFs that were responsible for regulating the expression of miR-675-3p in melanoma. To that end, we identified 32 shared TFs that were predicted to control the expression of this miRNA by both TransmiR and hTFtarget. We then constructed a comprehensive up- and downstream regulatory network pertaining to miR-675-3p that incorporated 32 TFs and 10 target genes. GO annotation analyses revealed that these TFs and genes were associated with key oncogenic processes, while KEGG pathway analyses suggested that miR-675-3p may regulate the cell cycle, transcriptional misregulation in cancer, TGF-beta, and HIF-1 signaling pathways. Flow cytometry and CCK8 assays revealed that miR-675-3p promotes cell cycle transition and proliferation, while Western blotting revealed that miR-675-3p mimic transfection may activate TGF-beta and HIF-1 signaling. Together, these findings thus emphasized the key role of miR-675-3p in the development and progression of melanoma.

In summary, our results indicated that miR-675-3p is upregulated in melanoma cell lines, tissues, and blood. Comprehensive up- and downstream regulatory network analyses demonstrated that miR-675-3p regulated numerous biological pathways in melanoma, making it a promising prognostic biomarker in patients with this form of cancer.

## Supplementary Information

Below is the link to the electronic supplementary material.

Supplementary file1 Supplementary figure 1 Pearson association between CG locus methylation level and miR-675-3p expression (TIF 18136 KB)
