# Supplementary figures and images for: Comprehensive upstream and downstream regulatory analyses identify miR-675-3p as a potential prognostic biomarker in melanoma
Source: Hum Cell. 2021 Jan 5;34(2):654–66. doi: 10.1007/s13577-020-00473-0 (PMC7900067; doi:10.1007/s13577-020-00473-0)

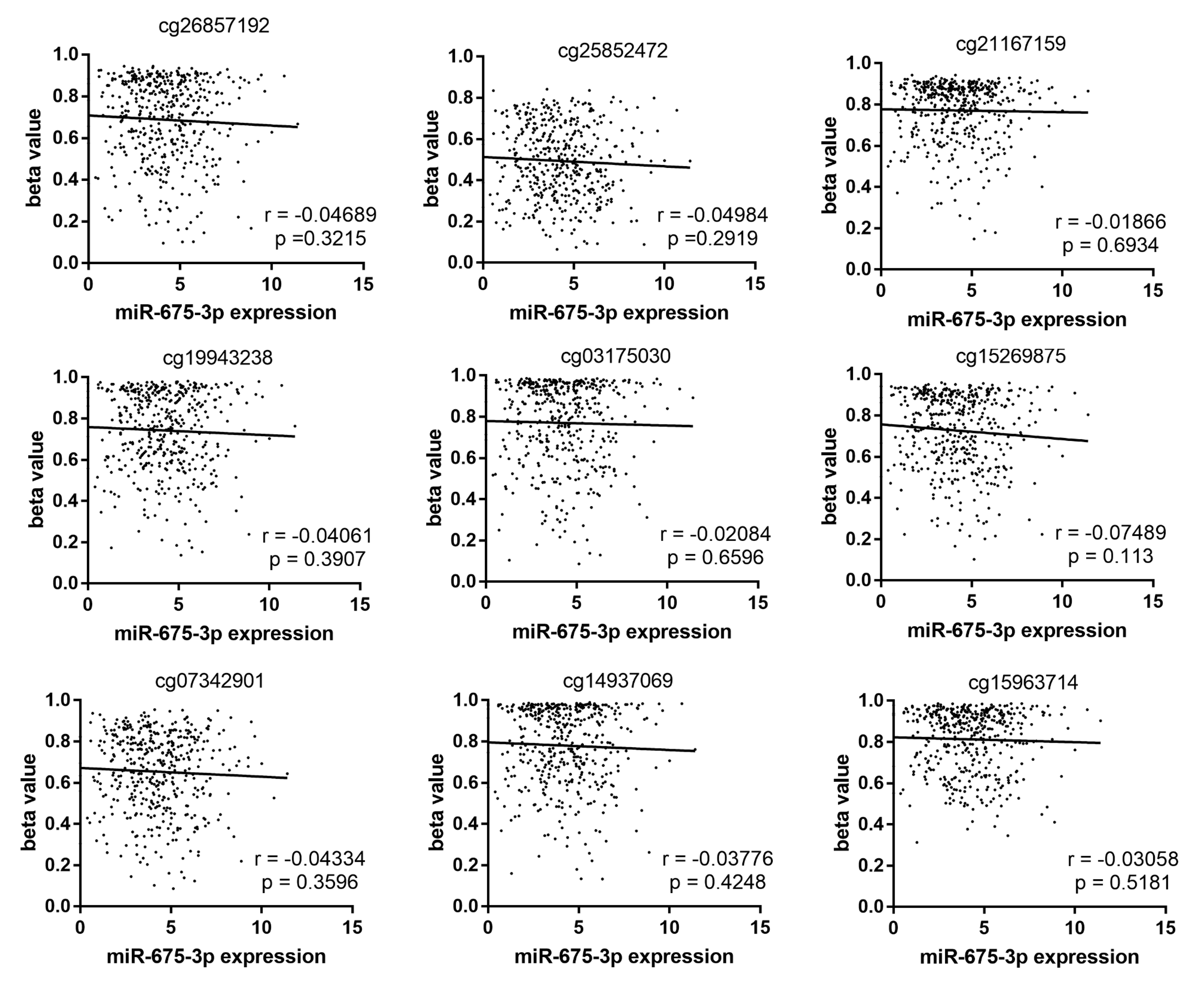

Supplement: Supplementary file 1 — Supplementary file1 Supplementary figure 1 Pearson association between CG locus methylation level and miR-675-3p expression (TIF 18136 KB) [file 13577_2020_473_MOESM1_ESM.tif]
